# Identification of a Novel Inhibitor of TfR1 from Designed and Synthesized Muriceidine A Derivatives

**DOI:** 10.3390/antiox11050834

**Published:** 2022-04-25

**Authors:** Yu Wu, Zongchen Ma, Xiaoyuan Mai, Xiaoling Liu, Pinglin Li, Xin Qi, Guoqiang Li, Jing Li

**Affiliations:** 1 Key Laboratory of Marine Drugs, Chinese Ministry of Education, School of Medicine and Pharmacy, Ocean University of China, Qingdao 266003, China; wuyu@stu.ouc.edu.cn (Y.W.); mazongchen@stu.ouc.edu.cn (Z.M.); maixiaoyuan@outlook.com (X.M.); liuxiaoling_1014@163.com (X.L.); lipinglin@ouc.edu.cn (P.L.); qixin_ouc@ouc.edu.cn (X.Q.); 2 Laboratory for Marine Drugs and Bioproducts, Open Studio for Druggability Research of Marine Natural Products, Qingdao National Laboratory for Marine Science and Technology, Qingdao 266237, China

**Keywords:** Muriceidine A, structural optimization, transferrin receptor 1, iron deprivation, anticancer agent

## Abstract

The transferrin receptor 1 (TfR1) plays a key role in cellular iron uptake through its interaction with iron-bound Tf. TfR1 is often reported to be overexpressed in malignant cells, and this increase may be associated with poor prognosis in different types of cancer, which makes it an attractive target for antitumor therapy. The marine natural product Muriceidine A is a potent anticancer agent reported in our previous work. In this study, we designed and synthesized a series of Muriceidine A derivatives and described the systematic investigation into their cytotoxic activities against four tumor cells. Most of the derivatives showed stronger antitumor activity and we found that the introduction of electron-donating groups at position C-2 of unsaturated piperidine was beneficial to anticancer activity and unsaturated piperidine was responsible for the antiproliferative activity. Among these compounds, **12b** (methyl at position C-2 of unsaturated piperidine) exhibited the strongest cytotoxicity against MDA-MB-231 cells. Further pharmacological research showed that **12b** bound to Transferrin receptor 1 (TfR1) directly caused iron deprivation and ROS imbalance along with the degradations of several oncoproteins, especially FGFR1, through the proteasome pathway; thus, inducing cell cycle arrest and apoptosis in MDA-MB-231 breast cancer cells. Our findings indicate that **12b** is a promising lead compound targeting TfR1 for triple negative breast cancer.

## 1. Introduction

Iron is an important element that plays a crucial role in various physiological and pathological processes in all kinds of mammalian cells [1,2]. It also has the capacity to engage in redox cycling and free radical formation. The balance between intracellular iron acquisition, storage and usage should be tightly regulated, since disorders of iron metabolism can induce tumorigenesis [3,4]. Iron may accelerate tumor initiation by enhancing the formation of free radicals, as well as function as a nutrient that fosters tumor cell proliferation. Compared with non-malignant cells, the growth of cancer cells is more dependent on iron and more sensitive to iron deprivation, a phenomenon known as “iron addiction” [5]. Evidence shows iron deprivation can induce apoptosis and autophagy in a series of cell lines, especially in breast cancer and hepatocellular carcinoma cells [6,7], suggesting that disruption of iron homeostasis is a promising strategy for cancer therapy investigation [8,9].

The transferrin receptor 1 (TfR1), also known as cluster of differentiation 71 (CD71), is a type II transmembrane glycoprotein that binds transferrin (Tf) and performs a critical role in cellular iron uptake and cellular iron homeostasis through the interaction with iron-bound Tf [10,11]. In general, TfR1 is expressed at low levels in most normal cells [12]. Many types of cancer cells reprogramme iron metabolism in ways that result in net iron influx. They upregulate proteins that are involved in iron uptake, such as TfR1, and decrease the expression of iron efflux proteins. Increased expression is observed in malignant cells with a high rate of proliferation and high need for iron, including breast [13], colon [14], liver [15], and brain cancer cells. In particular, cancer cells resistant to conventional therapies might particularly over-express TfR1 [16], indicating a poor prognosis for patients [17]. Interestingly, TfR1 can support mitochondrial respiration and reactive oxygen species production, both of which are key players in tumor cell growth and survival [18]. Taken together, the high expression of TfR1 on malignant cells, and its key role in cancer cell iron metabolism make it an attractive target for cancer therapy.

As TfR1 is an appealing therapeutic target for a variety of cancers, inhibition of TfR1 function by existing anti-TfR1 antibodies can lead to iron deprivation, which in turn promotes cancer cell death. The murine anti-human TfR1 IgG antibody 7579 downregulates TfR1 surface levels on cancer cells and has shown inhibitory effects on proliferation as well as inducing apoptosis of human U87MG, U251, and A172 glioma cells in vitro [19]; JST-TFR09, an antibody to human TfR1, has a great affinity to TfR1 in adult T-cell leukemia/lymphoma cells. It could interfere with the binding between TfR1 and Tf and inhibited the iron intake of adult T-cell leukemia/lymphoma, which has been used in preclinical development [20]. In addition, miRNA drug targeting TfR1 has been reported to be a good drug in the clinical treatment of leukemia [21]. However, all these reports about targeting TfR1 mainly focus on biopharmaceutical development. Compared to biological drugs, small molecule drugs have many more advantages, such as good permeability, low immunogenicity, cheap, easy storage, and transportation. However, there is still no small molecule compound reported to target TfR1 [22]. This underlines the significance to search for new TfR1 chemotherapeutic compounds with anticancer effects.

Marine natural products (MNPs) are regarded as important sources for the discovery of lead compounds for anti-cancer drugs, due to their unique chemical structures and diverse biological activities [23]. Simplifying complex structures of natural products, while retaining the desired biological activity, is a valid and meaningful strategy in the drug development process [24,25]. This strategy has been successfully used in the lead optimization of MNPs and yielded a number of marketed drugs and drug candidates [26,27].

**Figure 1:**
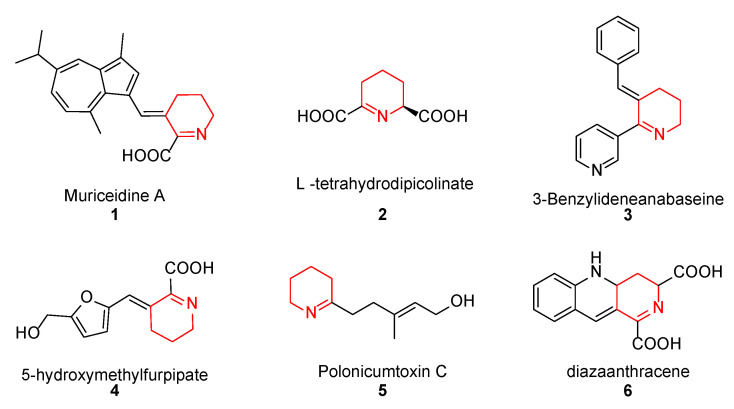
Chemical structure of some unsaturated piperidine derivatives.

Muriceidine A (**1**, Figure 1), a new skeleton guaiazulene alkaloid originally isolated from the South China Sea gorgonian *Muriceides collaris*, exhibits potent cytotoxic activity against the tumor cell line K562 with an IC_50_ value of 8.37 μM [28]. Structurally, Muriceidine A is comprised of the conjunction of guaiazulene and unsaturated pipecolic acid (Δ^1^-pipecolic acid). Guaiazulene (GA) has unique physical and chemical properties due to its electronic distribution and appearance features [29]. GA has potential research value for its anti-inflammatory, anti-allergy, anti-cancer, and other activities in the field of medicinal chemistry [30]. Δ^1^-pipecolic acid and its substitution derivatives are considered privileged scaffolds in drug discovery and are widely introduced in many antitumor drugs (**2**–**6**, Figure 1) [31,32,33]. Therefore, the modification of antitumor pharmacy cores with unsaturated piperidine structures has attracted much attention among medicinal chemists.

**Figure 2:**
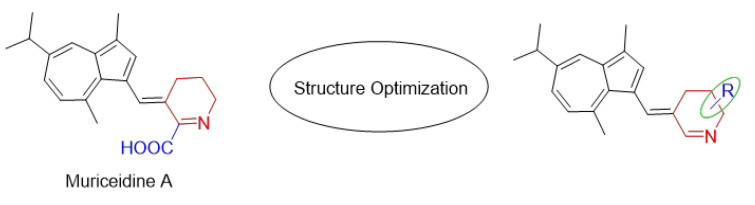
Chemical structure of Muriceidine A and designed compounds.

Encouraged by the antitumor activity of Muriceidine A and the virtues of unsaturated piperidine structures, it can be expected that the combination of unsaturated piperidine derivatives and guaiazulene may improve antitumor efficiency. Therefore, different substituent groups were introduced into the functional unsaturated piperidine moiety in the present work in the hope of obtaining agents with stronger antitumor activity (Figure 2). Herein, we describe the synthesis of five Muriceidine A derivatives (**12a**–**12d**, **16**) and the systematic investigation into their cytotoxic activities against four tumor cells (MDA-MB-231, K562, Hela, and HCT-116). Moreover, the antitumor mechanisms of the most active derivative, **12b**, were examined. It directly binds to TfR1, causing iron deprivation and ROS imbalance with degradations of several oncoproteins, especially FGFR1, through the proteasome pathway, thereby promoting cell apoptosis in MDA-MB-231 breast cancer cells.

## 2. Materials and Methods

### 2.1. Chemistry

#### 2.1.1. Materials and Methods

All reagents and solvents were commercially available. The structures of target compounds were confirmed by characterization with nuclear magnetic resonance (^1^H NMR and ^13^C NMR) and liquid chromatography-mass spectrometry (LC-MS). NMR spectra were recorded on the Bruker AVANCE instrument (300 MHz) at 25 °C using TMS as the internal standard in CDCl_3_. NMR spectra was shown in supplementary data (Figures S1–S11). Mass spectra were obtained using a Waters ZQ 2000 (Waters Instruments, Wilmslow, UK) electrospray ionization (ESI) single quadrupole mass spectrometer. Compounds were performed by flash column chromatography on silica gel (200–300 mesh) produced by Qingdao Marine Chemical Factory, Qingdao (China). Analytical thin-layer chromatography (TLC) was conducted on Fluka TLC plates (silica gel 60 F254, aluminum foil).

#### 2.1.2. General Procedure A for the Synthesis of Intermediates **11a**–**e**

T-Butyl hypochlorite (t-BuOCl): In a 25 mL round-bottom flask, t-BuOH (2 mL, 20.9 mmol), aqueous NaOCl solution (active chlorine ≥ 5%, 12 mL, 18.6 mmol), and acetic acid (2 mL, 34.3 mmol) were added in order and stirred at 0 °C for 5 min. The desired product was isolated from the lower aqueous phase as a yellow liquid. The organic fraction was directly used as a chlorine source in the next step without further purification.

Δ^1^-piperidine derivatives (**11a**–**e**) [34]: Compounds **9a**–**e** (2.1 mmol) were dissolved in a 50 mL flask with 15 mL of anhydrous ether, respectively. The freshly prepared t-BuOCl was then added dropwise at 0 °C under an N_2_ atmosphere. The resulting mixture was stirred at 0 °C for 3 h to give **10a**–**e**. TLC and ninhydrin colorimetry methods were used to track the reaction. When the reactants are completely converted, excessive solid KOH (500 mg, 8.9 mmol) was added in one batch, followed by 10 mL dry MeOH. The mixture was heated in an oil bath at 50 °C for 1h and then kept at room temperature overnight. The ether was removed by rotary evaporation at 20 °C which was further filtered to remove the solid and get the methanol solution of compounds **11a**–**e** (Δ^1^-piperidine derivatives). TLC proved that compounds **11a**–**e** existed as a near equimolar tautomeric mixture of enamine and imine. Due to the unstable nature of **11a**–**e**, the obtained solution was used in the next step without further purification.

#### 2.1.3. General Procedure B for the Synthesis of Final Products **12a**–**e**

Since intermediate **8** can efficiently be synthesized from commercially available guaiazulene **7** by a Vilsmeier-Hacck reaction [35], the synthesis process of **8** will not be described in detail here. CH_3_ONa (93 mg, 0.29 mmol) was dissolved in methanol (5 mL) in a 100 mL flask. Then, the methanol solution of compound **11a**–**e** (1 mmol) was added. The solution was stirred at room temperature for 5 min and then a 2 mL methanol solution of compound **8** (1 mmol) containing acetic acid (72 μL, 1.2 mmol) was added, and the mixture was added to the flask dropwise. Then the reaction was heated to 60 °C and stirred for 20 h. After cooling, the solution was evaporated to remove the methanol. The residue was dissolved in distilled water and extracted three times with EtOAc. The organic layers were combined and concentrated by evaporation to give the crude product which was further purified by column chromatography (eluent: CH_2_Cl_2_/CH_3_OH) to obtain the final products **12a**–**e**.

##### 3-((Guaiazulene-1-yl)ethylene) Δ^1^-piperidine (**12a**)

Following general procedure A and B, the crude residue obtained from piperidine was purified by flash-chromatography, using CH_2_Cl_2_/CH_3_OH 30:1 (*v/v*) as eluent, to furnish compound **12a** as a red solid (yield 50%). ^1^H NMR (500 MHz, CDCl_3_): δ 8.39 (s, 1H), 8.33 (s, 1H), 8.23 (s, 1H), 7.87 (s, 1H), 7.63 (d, *J* = 11.0 Hz, 1H), 7.44 (d, *J* = 11.0 Hz, 1H), 3.70 (s, 2H), 3.46 (s, 1H), 3.19–3.11 (m, 4H), 2.94 (t, *J* = 5.8 Hz, 2H), 2.58 (s, 3H), 2.07 (d, *J* = 5.7 Hz, 2H), 1.38 (d, *J* = 6.8 Hz, 6H). ^13^C NMR (126 MHz, CDCl_3_): δ 165.88, 150.80, 149.10, 148.66, 144.28, 141.80, 141.70, 139.07, 137.75, 135.35, 134.68, 129.58, 123.29, 119.79, 77.33, 77.08, 76.82, 42.50, 38.13, 29.44, 24.36, 23.83, 20.00, 13.17. MS (ESI): [M + H]^+^ 392.44.

##### 3-((Guaiazulene-1-yl)ethylene)-2-(methyl) Δ^1^-piperidine (**12b**)

Following general procedure A and B, the crude residue obtained from 2-methylpiperidine was purified by flash-chromatography, using CH_2_Cl_2_/CH_3_OH 30:1 (*v/v*) as eluent, to furnish compound **12b** as a red solid (yield 35%). ^1^H NMR (500 MHz, CDCl_3_) δ: 8.53 (s, 1H), 8.42 (s, 3H), 8.21 (d, *J* = 1.7 Hz, 1H), 7.80 (s, 1H), 7.57 (dd, *J* = 10.9, 1.7 Hz, 1H), 7.34 (d, *J* = 11.0 Hz, 1H), 3.71 (t, *J* = 5.3 Hz, 2H), 3.13 (dt, *J* = 13.7, 6.8 Hz, 1H), 3.07 (s, 3H), 2.93 (t, *J* = 5.9 Hz, 2H), 2.63 (s, 3H), 2.59 (s, 3H), 2.01 (dt, *J* = 11.9, 6.0 Hz, 2H), 1.37 (d, *J* = 6.9 Hz, 6H). ^13^C NMR (126 MHz, CDCl_3_): δ 173.26, 166.19, 147.45, 147.26, 143.73, 142.90, 140.54, 138.96, 137.32, 135.20, 133.10, 128.54, 122.03, 120.01, 77.37, 77.12, 76.86, 43.41, 38.04, 29.21, 24.50, 24.39, 20.29, 19.77, 13.13. MS (ESI): [M + H]^+^ 306.35.

##### 3-((Guaiazulene-1-yl)ethylene)-4-(hydroxymethyl) Δ^1^-piperidine (**12c**)

Following general procedure A and B, the crude residue obtained from 4-methanolpiperidine was purified by flash-chromatography, using CH_2_Cl_2_/CH_3_OH 20:1 (*v/v*) as eluent, to furnish compound **12c** as a red solid (yield 25%). ^1^H NMR (500 MHz, CDCl3) δ: 8.22 (s, 1H), 8.20 (d, *J* = 1.6 Hz, 1H), 8.09 (s, 1H), 7.57 (d, *J* = 10.9 Hz, 1H), 7.35 (d, *J* = 10.9 Hz, 1H), 3.89 (dd, *J* = 11.0, 3.4 Hz, 1H), 3.85–3.69 (m, 4H), 3.64 (s, 1H), 3.13 (dt, *J* = 13.8, 6.8 Hz, 1H), 3.06 (s, 3H), 2.59 (s, 3H), 2.45 (d, *J* = 12.8 Hz, 1H), 1.86 (s, 1H), 1.38 (d, *J* = 6.9 Hz, 6H). ^13^C NMR (126 MHz, CDCl_3_): δ 166.09, 149.34, 148.43, 147.97, 143.96, 141.74, 138.48, 137.30, 135.10, 134.28, 130.06, 122.33, 120.74, 77.27, 77.02, 76.76, 61.01, 39.37, 38.04, 34.29, 29.57, 24.36, 21.15, 13.21. MS (ESI): [M + H]^+^ 321.66.

##### 3-((Guaiazulene-1-yl)ethylene)-Δ^1^-pyrrolidine (**12d**)

Following general procedure A and B, the crude residue obtained from pyrrole was purified by flash-chromatography, using CH_2_Cl_2_/CH_3_OH 15:1 (*v/v*) as eluent, to furnish compound **12d** as a red solid (yield 6%). ^1^H NMR (500 MHz, CDCl_3_): δ 8.61 (d, *J* = 12.0 Hz, 2H), 8.24 (s, 3H), 7.83 (s, 1H), 7.62 (d, *J* = 11.4 Hz, 1H), 7.41 (d, *J* = 10.9 Hz, 1H), 4.33–4.28 (m, 2H), 3.32–3.26 (m, 3H), 3.20–3.12 (m, 4H), 2.60 (s, 3H), 1.39 (d, *J* = 6.9 Hz, 6H). ^13^C NMR (126 MHz, CDCl_3_): δ 170.56, 140.18, 137.85, 137.49, 135.33, 134.06, 52.36, 38.09, 29.28, 27.70, 24.39, 13.23. MS (ESI): [M + H]^+^ 277.42.

##### Muriceidine A (**12e**)

Following general procedure A and B, the crude residue obtained from L-pipecolic acid was purified by flash-chromatography, using CH_2_Cl_2_/CH_3_OH 15:1 (*v/v*) as eluent, to furnish compound Muriceidine A as a red solid (yield 10%). ^1^H NMR (500 MHz, CDCl_3_) δ: 9.57 (s, 1H), 8.15 (s, 1H), 7.77 (s, 1H), 7.50 (d, *J* = 10.7 Hz, 1H), 7.32 (d, *J* = 10.7 Hz, 1H), 3.71 (s, 2H), 3.15 (s, 3H), 3.10 (m, 1H), 2.88 (s, 2H), 2.57 (s, 3H), 1.99 (s, 2H), 1.37 (d, *J* = 6.9 Hz, 6H). MS (ESI): [M + H]^+^ 336.21.

#### 2.1.4. Synthesis of 5-((Guaiazulene-1-yl)ethylene)-1,3-dimethylhydantoin (**16**)

Intermediate 1,3-dimethylhydantoin (**15**) was obtained from glyoxal (**13**) and 1,3-dimethylurea (**14**) according to the method described in the literature [34]. Product **16** was then synthesized using the same method as procedure B, by reacting **15** with 3-formyl guaiazulene **8**. The crude residue was purified by flash-chromatography, using CH_2_Cl_2_/CH_3_OH 15:1 (*v/v*) as eluent, to furnish compound **16** as a green solid (yield 24%). ^1^H NMR (600 MHz, CDCl_3_) δ 8.13 (d, *J* = 1.9 Hz, 1H), 7.60 (s, 1H), 7.42 (s, 1H), 7.39 (dd, *J* = 10.7, 1.8 Hz, 1H), 7.02 (d, *J* = 10.8 Hz, 1H), 3.16 (s, 3H), 3.05 (dt, *J* = 13.8, 6.9 Hz, 1H), 3.00 (s, 3H), 2.95 (s, 3H), 2.62 (s, 3H), 1.37 (d, *J* = 6.9 Hz, 7H). ^13^C NMR (151 MHz, CDCl_3_) δ 164.1, 156.4, 147.0, 142.1, 139.4, 139.4, 136.3, 136.0, 134.60, 128.4, 126.9, 125.1, 118.4, 113.3, 77.4, 77.2, 77.0, 38.0, 30.8, 27.7, 25.1, 24.6, 13.0. MS (ESI): [M + H]^+^ 337.52.

### 2.2. Biological Evaluation

#### 2.2.1. Cell Culture and Reagents

All human cancer cell lines were provided by the Institute of Biochemistry and Cell Biology, Chinese Academy of Sciences (Shanghai, China). The human triple negative breast cancer cell line MDA-MB-231 was cultured in L-15 complete medium (Gibco, Grand Island, NY, USA). SH-SY5Y, Siha, PC-3, HO-8910, MGC-803, NCI-H1975, and BEL-7402 cell lines were cultured in RPMI-1640 medium (Gibco, Grand Island, NY, USA). HeLa and MCF-7 cell lines were cultured in MEM medium. A549 cell line was cultured in F-12K medium (Gino, Hangzhou, China). The K562 cell line was cultured in IMDM medium (Gibco, Grand Island, NY, USA). The HCT116 cell line was cultured in high glucose DMEM medium (Gibco, Grand Island, NY, USA). Cell lines were supplemented with 10% FBS (Gibco, Grand Island, NY, USA), 2 mM L-glutamine, and 1% penicillin-streptomycin (Solarbio, Beijing, China) and propagated as monolayer cultures at 37 °C in a humidified 5% CO_2_ incubator.

#### 2.2.2. Cell Viability Assay

The inhibition of cancer cell viability was assessed by the MTT assay. Cells were plated into a 96-well plate at a density of 5 × 10^3^ cells per well. After attachment, cells were treated with **12b** in different concentrations or 1% DMSO solution as the negative control group. After 72 h, 20 μL of MTT (Sigma-Aldrich, St. Louis, MO, USA) was added to each well. Four hours later, the formazan product was dissolved in DMSO and quantitated spectrophotometrically at a wavelength of 570 nm using a microplate reader (BioTek, Winooski, VT, USA). The IC_50_ value was defined as the concentration that inhibited cell viability by 50%.

#### 2.2.3. Flow Cytometry Analysis of Cell Cycle

MDA-MB-231 cells were plated into 6-well plates at a density of 2.5 × 10^5^ cells per well and were treated with **12b** at indicated concentrations for 24 h. After that, the cells were collected and washed in PBS and fixed in ice-cold 70% (*v/v*) ethanol overnight. After washing twice with PBS, cells were stained with a solution containing 50 μg/mL PI (Sigma, St. Louis, MO, USA) and 10 μg/mL RNase A (Solarbio, Beijing, China) for 30 min in the dark at 4 °C. Cell cycle distribution analysis was performed using a MoFlo XDP flow cytometry system (Beckman Coulter, Boulevard Brea, CA, USA).

#### 2.2.4. Extraction and Western Blotting

MDA-MB-231 cells were incubated with **12b** at indicated concentrations, then washed with PBS and disrupted on ice for 40 min by a loading buffer, and boiled for 15 min.

The protein concentration of lysates was quantified by using a BCA Protein Assay Kit (Beyotime Biotechnology, Shanghai, China). Equal amounts of protein were separated using sodium dodecyl sulfate (SDS)-polyacrylamide gel electrophoresis (PAGE), and then transferred to nitrocellulose membranes. The membranes were blotted with primary antibodies overnight at 4 °C followed by HRP-conjugated secondary antibodies for 1 h at room temperature. Proteins on the membranes were visualized with enhanced chemiluminescence by using chemiluminescence detection reagents.

Antibodies to detect Cleaved poly-(ADP-ribose) polymerase (C-PARP), Caspase 8, Cleaved Caspase 3 (C-Cas3), Survivin, γ-H2AX, Bcl-2, Mcl-1, P21, Akt, phosphor-Akt (Ser473), ERK1/2, phosphor-ERK1/2 (pT185/pY187), Stat3, phosphor-Stat3 (Ser727), PDGFR, FGFR1, VEGFR, EGFR, Src, and phosphor-Src (Tyr416) were purchased from Cell Signaling Technology (Boston, MA, USA). TfR1 antibody, the primary antibody β-Tubulin, and the secondary antibodies were purchased from Sangon Biotech (Shanghai, China).

#### 2.2.5. Immunofluorescence

MDA-MB-231 cells were seeded in 384-well microplates with a glass bottom (Corning, Corning, NY, USA) at a density of 1 × 10^3^ cells per well. After being treated with **12b** at 4 μmol/L for 24 h, the cells were fixed with 4% paraformaldehyde (Sangon Biotech, Shanghai, China) in PBS for 30 min and permeabilized with 0.3% Triton X-100 (Sangon Biotech, Shanghai, China) for 15 min at room temperature. After blocking with 1% BSA in PBS, the cells were stained overnight with primary antibody FGFR1 in blocking buffer at 4 °C, then incubated with secondary FITC conjugated anti-rabbit IgG antibody (Solarbio, Beijing, China) for 1 h at room temperature. 4′,6-diamidino-2-phenylindole (DAPI, Solarbio Science, Beijing, China) was used for nuclear staining. The resulting images were captured using a laser scanning confocal microscope (Carl Zeiss, Jena, Germany).

#### 2.2.6. Cellular Thermal Shift Assay (CETSA)

MDA-MB-231 cells were plated into 6-well plates at a density of 2.5 × 10^5^ cells per well. After attaching overnight, cells were treated with **12b** at 4 μmol/L for 2 h. Cells were harvested and resuspended in PBS and digested with 0.25% trypsin (Sigma-Aldrich, St. Louis, MO, USA). The cells were then resuspended with PBS and divided into several PCR tubes. Six aliquots were treated with DMSO and another six aliquots were treated with **12b**. The tubes were heated at different temperatures by a PCR Thermal Cycler Dice (Analytikjena, Germany) for 3 min and then cooled at 25 °C for 3 min. The heated cells were treated with protease inhibitor PMSF (Solarbio, Beijing, China) and were freeze-thawed three times using liquid nitrogen to lyse cells. Cell lysates were centrifuged at 20,000× *g* at 4 °C for 20 min. The supernatants were harvested and loading buffer was added before boiling. Protein levels were assessed by Western blotting.

#### 2.2.7. Ricinus Communis Agglutinin I (RCA I) Staining and Mass Spectrometric Analysis

Following the CETSA assay, equal amounts of protein were run on 8% SDS-PAGE gels, then transferred to nitrocellulose membranes. The membranes were incubated with the indicated RCA I antibody (Vector Laboratories, Inc., Burlingame, CA, USA) for 30 min at room temperature, followed by HRP-Streptavidin antibody (R&D Systems, Minneapolis, MN, USA) at room temperature for 1 h. After washing with TBST, signals were detected by chemiluminescence with the enhanced chemiluminescence (ECL) detection reagents.

The gel strips around 80 KD were cut and sent to OE Biotech (Shanghai, China). The **12b**-binding proteins were detected by liquid chromatography-mass spectrometry/mass spectrometry. The **12b**-interacting candidate proteins were identified and analyzed by using Mascot 2.3 software (Matrix Science, Chicago, IL, USA) and the UniProt-Homo database.

#### 2.2.8. SiRNA and Transfections

MDA-MB-231 cells were plated into 6-well plates at a density of 2.5 × 10^5^ cells per well and were transfected at 70–90% confluency with 100 nM Si-NC or Si-TfR1 (sense: 5′-GGCCAGCAAAGUUGAGAAATT-3′) using Lipofectamine 3000 Transfection Reagent (Thermofisher, Waltham, MA, USA) according to the manufacturer’s instructions. Small interfering RNAs (siRNAs) were purchased from Gene Pharma. Western blotting was used to detect the knockout efficiency of target genes 48 h later.

#### 2.2.9. Detection of Cellular Fe^2+^ Ions Generation

To clarify Fe^2+^ ions generation via **12b** treatment, CLSM measurement was performed. MDA-MB-231 cells were seeded in 24-well plates at the density of 5 × 10^4^ per well, cultured for 24 h, and then **12b** (2, 4 μM) was added into each well, respectively. Meanwhile, DMSO was prepared as a control group. After 24 h, the medium was removed and cells were washed with PBS three times. Then the intracellular Fe^2+^ ions probe FerroOrange was diluted to 1 μM in serum-free medium and was added to the cells. Cells were incubated at 37 °C for 30 min in the dark. Finally, the fluorescence images of cells were captured by using a confocal microscope (Ex: 543 nm, Em: 580 nm). FerroOrange was purchased from the Dojindo Molecular Technologies Company.

#### 2.2.10. Reactive Oxygen Species (ROS) Level Detection

ROS levels were detected using 2,7-dichloro-dihydrofluorescein diacetate (H_2_DCFDA) (Biosharp Life Science, Hefei, China) and dihydroethidium (DHE) (Sigma-Aldrich). MDA-MB-231 cells were seeded in 24-well plates at the density of 5 × 10^4^ per well, cultured for 24 h, and then treated with **12b** at indicated concentrations. Resup (50 mg/mL) was used as a positive control group. After being cultured for 24 h, the cells were washed with PBS three times. Then, H_2_DCFDA as an intracellular ROS ion probe (10 μM) was added to the cells. The cells were stationary cultured at room temperature in the dark. Fluorescence images of cells were presented using confocal microscopy (Ex: 488 nm, Em: 530 nm). After **12b** treatment for 24 h, cells were harvested and incubated with DHE (5 μM) for 30 min at 37 °C in the dark. DHE fluorescence (Ex: 535 nm, Em: 610 nm) was measured using a MoFlo XDP flow cytometry system (Beckman Coulter, Boulevard Brea, CA, USA).

#### 2.2.11. Statistical Analysis

Statistical analyses were performed using the GraphPad Prism 5 software (GraphPad Inc, San Diego, CA, USA). Data were presented as mean values ± standard deviation. Statistical significance was analyzed by one-way ANOVA for at least three independent experiments. A *p*-value < 0.05 was considered statistically significant.

## 3. Results

### 3.1. Chemistry

**Scheme 1:**
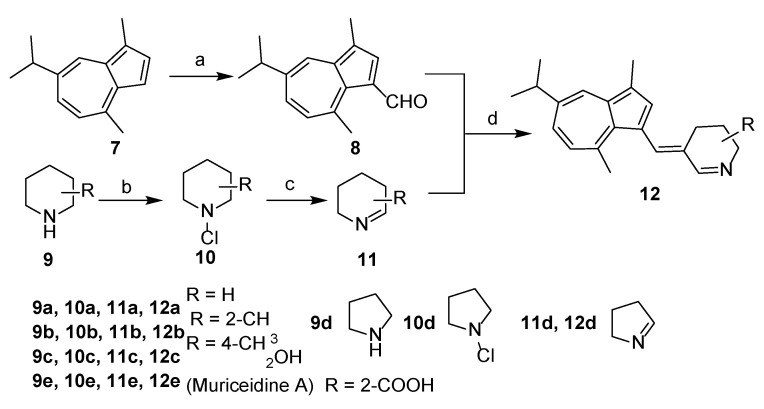
Synthesis of **12a**–**e**. Reagents and conditions: (a) POCl_3_, DMF, 0 °C to rt, 1 h, 95%; (b) t-BuOCl, Et_2_O, 0 °C, 3 h; (c) KOH, Et_2_O, MeOH, 50 °C, 1 h, rt, 10h; (d) AcOH, CH_3_ONa, MeOH, 60 °C, 20 h, 8–20%.

**Scheme 2:**
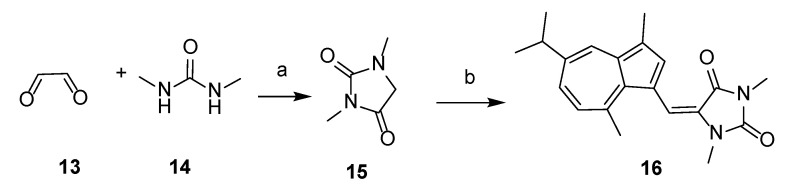
Synthesis of **16**. Reagents and conditions: (a) H_2_SO_4_, 100 °C, 1 h, 80%; (b) AcOH, CH_3_ONa, MeOH, compound **8**, 60 °C, 20 h, 24%.

The overall synthetic routes of the title compounds are illustrated in Scheme 1 and Scheme 2. 3-formyl guaiazulene **8** was efficiently synthesized from commercially available guaiazulene **7** following a Vilsmeier-Hacck reaction [35] (Scheme 1). The Δ^1^-piperidine derivatives **11a**–**e** were synthesized from piperidine derivatives **9a**–**e** through chlorination and dehydrochlorination, following our previously reported methods (Scheme 1). Under the presence of sodium methoxide and acetic acid, **12a**–**e** could be obtained by an aldol-condensation-like reaction between **8** and **11a**–**e** in an 8–20% yield. (Scheme 1). Compound **16** was obtained by condensation of 3-formyl guaiazulene **8** and 1,3-dimethylhydantoin **15** in 24% yield (Scheme 2) [36].

### 3.2. Biological Activity

#### 3.2.1. Antitumor Activity In Vitro

In this study, the MTT assay was used as a preliminary screen for the evaluation of in vitro cytotoxicity. The antitumor-viability effect of all target compounds (**12a**–**12d**, **16**) was evaluated against four cancer cell lines: MDA-MB-231, K562, Hela, and HCT-116. Doxorubicin was used as a positive control. The results were expressed as IC_50_ values and summarized in Table 1.

Generally, the antitumor activity of the tested compounds varied with different substitutions of Δ^1^-piperidine at different positions. Compounds (**12b** and **12c**) showed more potent inhibitory activity against cancer cells with IC_50_ values < 10 μM than that of other compounds. It is notable that compound **12b** was the most active derivative which showed potent activities against four cancer cell lines with IC_50_ values from 1.15 μM to 4.63 μM, and displayed the best activity against MDA-MB-231 cells with an IC_50_ value of 1.15 μM.

For the MDA-MB-231 cell line, compounds **12a** (Δ^1^-piperidine), **12b** (methyl at position C-2 of Δ^1^-piperidine), **12c** (hydroxymethyl at position C-4 of Δ^1^-piperidine), and **12d** (Δ^1^-pyrrolidine) showed higher inhibitory activities (IC_50_ = 13.93, 1.15, 4.64, and 10.91 μM, respectively) than Muriceidine A (IC_50_ = 77.61 μM). Among these compounds, compound **12b** with a methyl at position C-2 of Δ^1^-piperidine (IC_50_ = 1.15 μM) was the most active compound and was 67.5-fold more potent than Muriceidine A. For the K562 cell line, compounds **12a**, **12b**, and **12c** were the most active compounds with IC_50_ values of 6.99, 2.41, and 1.91 μM, respectively. Compound **12c** was approximately 4.4-fold more potent than Muriceidine A (IC_50_ = 8.37 μM). For the Hela cell line, compounds **12a**, **12b**, and **12c** exhibited significant cytotoxicity with IC_50_ values of 8.73, 1.21, and 2.58 μM, respectively. Notably, compound **12b** had 15-fold higher cytotoxic activity than Muriceidine A (IC_50_ = 14.9 μM). For the HCT-116 cell line, only compounds **12b** and **12c** exhibited significant cytotoxicity with IC_50_ values of 4.63 and 3.22 μM, respectively.

Taken together, in comparing the IC_50_ values, we found that the Δ^1^-piperidine derivatives with electron-donating groups (H/Me) at C-2 had a profound effect on antiproliferative activity since the potency increased following the order Me > H > COOH, which indicated that the combination of Δ^1^-piperidine derivatives with electron-donating groups and guaiazulene was beneficial for cytotoxic activities. The replacement of methanol at C-4 in Δ^1^-piperidine can also significantly enhance the anti-proliferative activity. Interestingly, compared with compound **12b** which possesses piperidine, compounds with pyrrolidine (**12d**) or 1,3-dimethylhydantoin (**16**) displayed decreased activity, suggesting that Δ^1^-piperidine was responsible for the antiproliferative activity.

#### 3.2.2. Compound **12b** Inhibits Various Cells’ Viability and Induces MDA-MB-231 Cells Apoptosis

**Figure 3:**
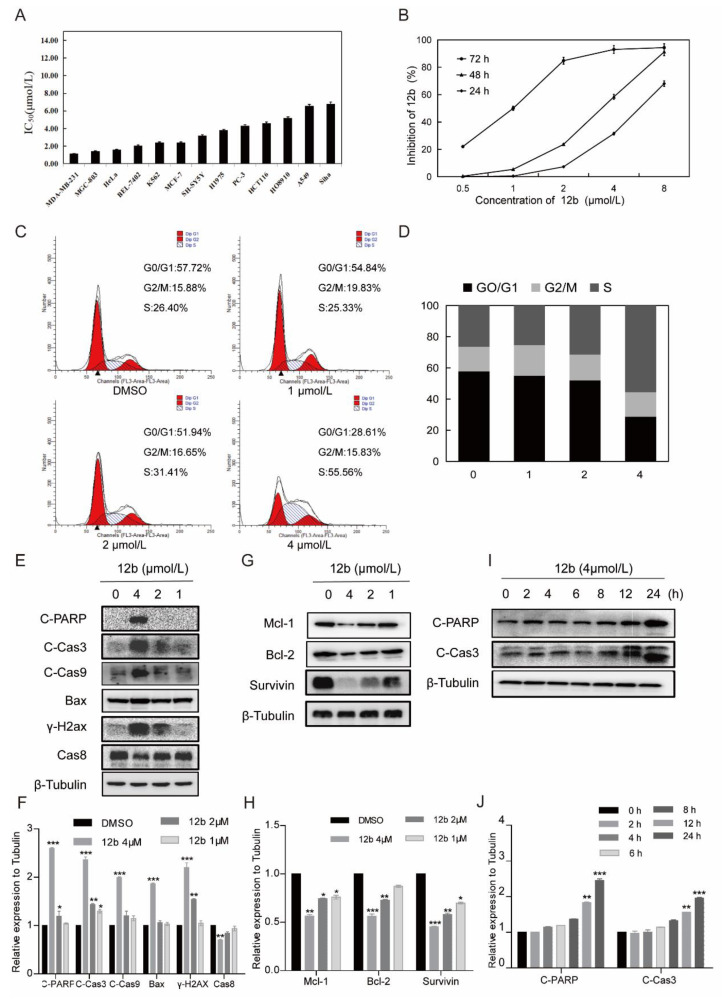
**12b** inhibits various cells’ viability and induces MDA-MB-231 cells apoptosis. (**A**) IC_50_ values of **12b** on various tumor cells. Cancer cells were treated with the indicated concentration of **12b** for 72 h. Cell proliferation was determined by using an MTT assay. (**B**) Proliferation inhibition of **12b** on MDA-MB-231 cells. Cells were treated with the indicated concentration of 1**2b** for 24, 48, and 72 h. Cell proliferations were determined by MTT assay. (**C**) **12b** affected cell cycle distribution. Cells were treated with **12b** (1–4 μM) for 24 h, then were collected and stained by PI. The DNA content of cells was determined with the MoFlo XDP flow cytometry system. (**D**) Histograms showed the percentage of cells in G0/G1, G2/M, and S phase after treatment with **12b**. (**E**–**J**) Effects of **12b** on the expression of proteins related to apoptosis. MDA-MB-231 cells were treated with **12b** (1, 2, 4 μM) for 24 h or treated with **12b** (4 μM) for indicated times. Protein levels were analyzed by Western blotting. The relative band intensities of proteins to the β-Tubulin were analyzed by Image J software. Protein band densities were quantified by normalizing to the DMSO group. Data are presented as means ± SD. All experiments were performed in three replicates. * *p* < 0.05, ** *p* < 0.01, *** *p* < 0.001.

As compound **12b** displayed the best antiproliferative activity in the initial screening, the inhibitory activity of **12b** on more types of human cancer cells was next to be examined. As shown in Figure 3A, **12b** exhibited cytotoxic IC_50_ values in the range of 1.15–7 μM after 72 h treatment in various cancer cells with the lowest one towards MDA-MB-231 cells, and dose- and time-dependently suppressed the viabilities of these cells (Figure 3B). Thus, in the following studies, MDA-MB-231 cells were used to further investigate the anticancer effect and mechanism of **12b**.

To investigate whether cell cycle arrest is associated with **12b**-induced viability inhibition, we further analyzed the cell cycle distribution and found that MDA-MB-231 cells in S phase increased from 26.4% to 55.56% in the presence of 4 μM **12b** (Figure 3C,D). These results suggest that compound **12b** inhibits the viability of cancer cells by arresting the cell cycle in the S phase.

Next, we determined whether S phase arrest evoked by **12b** resulted in cell apoptosis. Cysteine-aspartate-specific protease-9 (Caspase-9) and cysteine-aspartate-specific protease-3 (Caspase-3) are considered to be important components of the effector caspases. Moreover, cleavage and activation of caspase-9/caspase-3 are hallmarks of innate apoptosis [37]. In addition, poly (ADP-ribose) polymerase (PARP) is the substrate of Caspase-3, which has been reported as an early marker of apoptosis [38]. As shown in Figure 3E,I, the expression levels of the active cleaved fragments of Caspase-9, Caspase-3, and PARP were distinctly increased with **12b** treatment. Moreover, the level of caspase-8, an important component of the death signal receptor pathway of apoptosis [39], decreased after exposure to **12b** treatment, implying the activation of caspase-8. Bax, a member of the Bcl-2 family, can promote apoptosis mediated by mitochondria. We also observed obvious increases of Bax in the presence of **12b**. In addition, DNA strand breaks have been demonstrated to play a crucial role in regulating the apoptotic pathway [40]. The levels of γ-H2AX, an essential marker of DNA double-strand breaks [41], were increased significantly in a dose-dependent manner. Cell apoptosis occurs frequently by overexpression of inhibitor of apoptosis (IAP) and Bcl-2 family antiapoptotic proteins. The IAP family such as survivin, and the Bcl-2 family including Bcl-2 and Mcl-1 proteins play important roles in suppressing apoptosis of cells mediated by mitochondria [42]. We then tested whether **12b** affected the expression levels of these anti-apoptotic proteins. After treatment with 1 to 4 μM **12b** for 24 h, the protein levels of survivin, Bcl-2, and Mcl-1 were obviously decreased (Figure 3G). Taken together, **12b** induces cell apoptosis through the mitochondrial pathway combining with the death receptor pathway in MDA-MB-231 cells.

#### 3.2.3. Compound **12b** Induces Degradation of Receptor Protein in MDA-MB-231 Cells through the Proteasome Pathway

**Figure 4:**
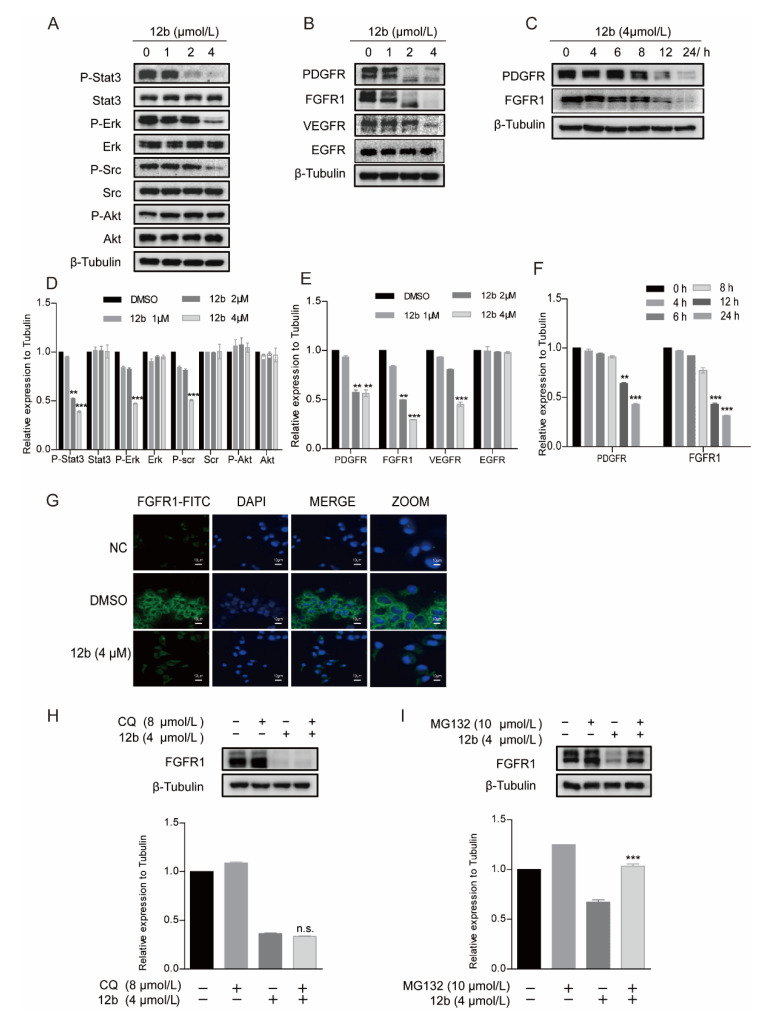
**12b** induces degradation of receptor protein in MDA-MB-231 cells through the proteasome pathway. (**A**) MDA-MB-231 cells were treated with **12b** (0–4 μM) for 24 h and the levels of proliferation-related proteins were detected by Western blotting. (**B**,**C**) MDA-MB-231 cells were treated with **12b** (0–4 μM) for 24 h or **12b** (4 μM) for the indicated times and the levels of tyrosine kinase receptors were detected by Western blotting. (**D**–**F**) The relative band intensities of proteins to the β-Tubulin were analyzed and densities were quantified by normalizing to the DMSO group. (**G**) MDA-MB-231 cells were treated with **12b** (4 μM) for 24 h and were stained with primary antibody FGFR1, followed by FITC-antibody. DAPI was used for nuclear staining. The confocal microscope was used for immunofluorescence analysis. The scale bars are 10 μm. (**H**) MDA-MB-231 cells were treated with **12b** (4 μM) or/and 8 μM Chloroquine for 12 h. (**I**) MDA-MB-231 cells were treated with **12b** (4 μM) or/and 10 μM MG132 for 12 h. Protein levels were examined by Western blotting. Protein band densities were quantified by normalizing to β-Tubulin (below). Data are presented as means ± SD. All experiments were performed in three replicates. ** *p* < 0.01, *** *p* < 0.001.

Cell apoptosis is regulated by cell signal transduction. To explore the mechanism underlying **12b**-mediated cytotoxicity, we examined major signaling pathways including the expression and activation of AKT [43], ERK [44], and STAT3 in MDA-MB-231 cells. As shown in Figure 4A, after treatment with 1, 2, and 4 μM **12b** for 24 h, the phosphorylation of STAT3, Src, and ERK decreased significantly. These results indicate that **12b**-induced cell apoptosis is probably associated with the inactivation of cell proliferation upstream signaling. Thus, we further examined the kinase expression and activities upon **12b** on some upstream tyrosine kinase receptors including PDGFR, FGFR1, VEGFR, and EGFR. The results show that the expression levels and activities of PDGFR, FGFR1, and VEGFR other than EGFR were reduced by **12b** in a time and dose-dependent manner in MDA-MB-231 cells (Figure 4B,C). Increasing evidence supports that FGFR1, a typical kind of receptor tyrosine kinase (RTK), can play a more important part in TNBC progression as the expression of FGFR1 is an independent negative prognostic factor in it [45,46,47]. As FGFR1 expressed on the cell membrane is closely related to the malignant biological behavior of breast cancer, an immunofluorescence assay was used to further determine FGFR1 levels. Compared with the control group, **12b** significantly reduced FGFR1 expression on the surface of cells (Figure 4G). These results suggest that **12b** can selectively inhibit the expressions of some kinase receptor proteins including FGFR1 in MDA-MB-231 cells.

We further explored the cause of the decrease in FGER1 protein as an example. As the reduction of protein level via degradation by lysosomal and proteasome is a common way, we first treated cells with protein lysosomal inhibitor Chloroquine before the addition of **12b** and found that Chloroquine did not significantly reverse the protein levels of FGFR1 reduced by **12b** (Figure 4H). Next, MG132 was used as a proteasome inhibitor to investigate the proteasome pathway. As shown in Figure 4I, we found significant suppressions of **12b**-induced FGFR1 degradation by MG132 in MDA-MB-231 cells. These results indicate that the **12b**-induced down-regulation of receptor proteins is related to proteasome degradation.

#### 3.2.4. Compound **12b** Can Bind to a Glycoprotein Containing α, β-D Galactose

**Figure 5:**
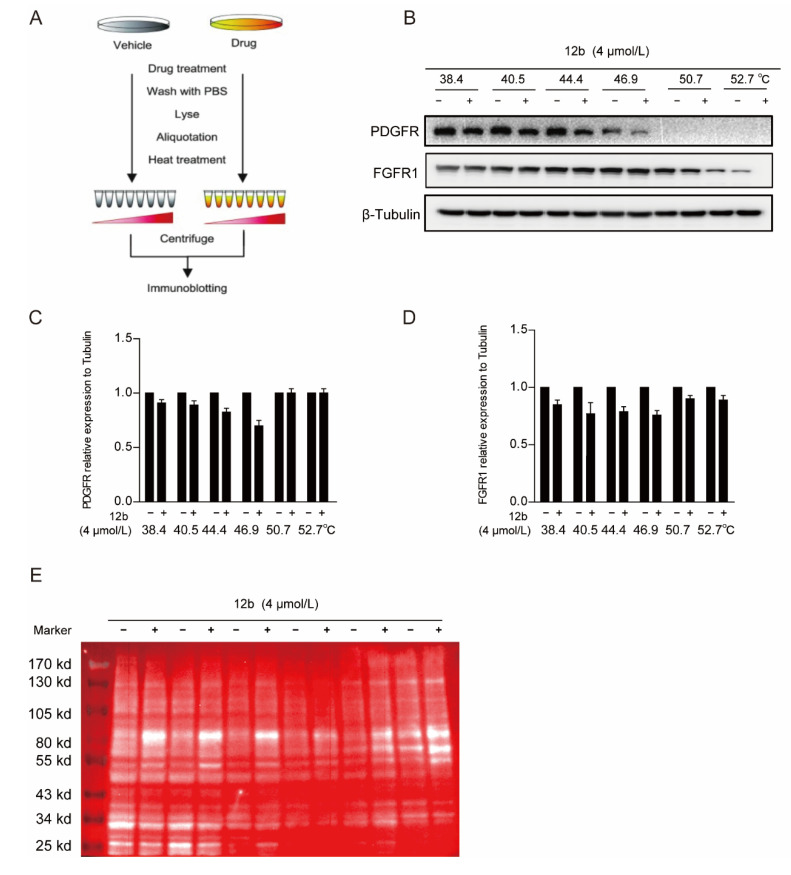
**12b** bind to a glycoprotein containing α, β-D galactose. (**A**,**B**) Effect of **12b** on RTK proteins stabilization. MDA-MB-231 cells were treated with or without **12b** (4 μM) for 2 h before being heated at different temperatures. Protein levels were analyzed by Western blotting. (**C**,**D**) The relative band intensity to β-Tubulin was analyzed by ImageJ software. Protein band densities were quantified by normalizing to DMSO. (**E**) The samples in (**A**) were taken for immunoblotting, membranes were incubated with the RCA I antibody, and HRP-Streptavidin antibody. Signals were detected by chemiluminescence.

We next performed a cellular thermal shift assay (CETSA) to determine whether **12b** reduced tyrosine kinase receptor expression of PDGFR and FGFR1 by binding to it directly in cells. CETSA is a method based on the ligand-induced thermal stability of protein to directly detect the interaction between a drug molecule and target protein (Figure 5A) [48]. We showed that FGFR1 and PDGFR were degraded with increased incubation temperature after **12b** treatment, suggesting that **12b** does not bind to the two tyrosine kinase receptors directly in MDA-MB-231 cells (Figure 5B–D).

Since **12b** significantly inhibited multiple downstream signals in cells, we still suspected that it was related to cellular receptor proteins. Membrane receptor proteins tend to be highly glycosylated, which is one of the most common post-translational modifications. Ricinus Communis Agglutinin I (RCA I) as a kind of lectin can specifically bind to α, β-D galactose, which are widely found in most glycoprotein chains. Thus, we used RCA I to screen the binding of glycoproteins to **12b** in MDA-MB-231 cells by a modified CESTA experiment and found that the glycoproteins around 80 KD were distinctly thermally stable after **12b** treatment. These results indicate that **12b** can bind to a glycoprotein containing α, β-D galactose around 80 KD (Figure 5E).

#### 3.2.5. Compound **12b** Disrupts the Iron Homeostasis and Cellular Oxidative Balance

To explore the **12b** binding glycoprotein, the gel strips around 80 KD were cut and analyzed by liquid chromatography-mass spectrometry/mass spectrometry. As shown in Table 2, among these candidates, TfR1 was the only receptor glycoprotein in a high percentage sequence coverage.

**Figure 6:**
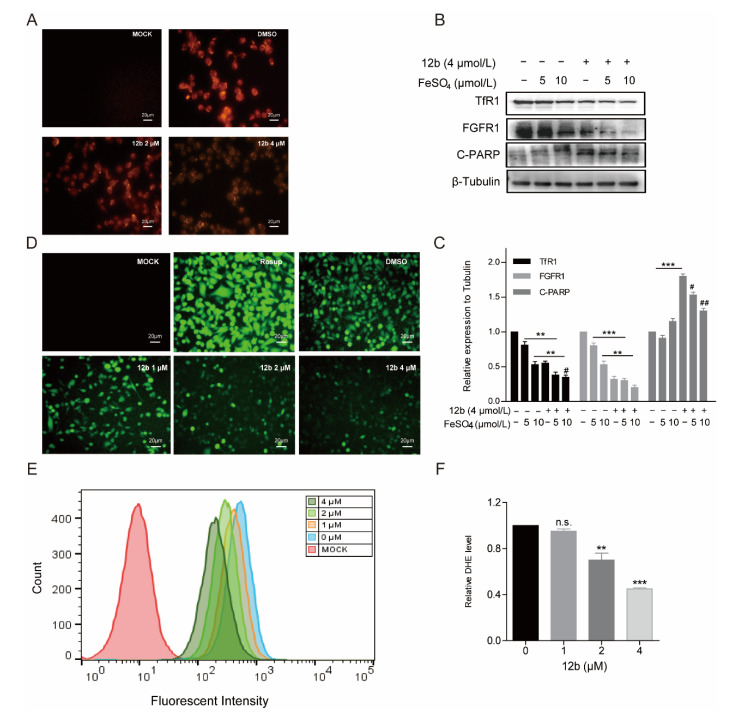
**12b** disrupts the cellular oxidative balance. (**A**) CLSM images of MDA-MB-231 cells were collected to visualize the intracellular Fe^2+^ ions generation using the Fe^2+^ ions fluorescent probe Ferrorange. The scale bars are 20 μm. (**B**) MDA-MB-231 cells were pretreated with FeSO_4_ (0, 5, 10 μM) for 24 h and treated with or without **12b** (4 μM). Protein levels were analyzed by Western blotting. (**C**) The protein band densities were quantified by normalizing to DMSO. (**D**) CLSM images of MDA-MB-231 cells treated under different conditions of **12b** to evaluate ROS production based on DCF fluorescence intensity using the fluorescent probe H_2_DCFDA. Resup (50 mg/mL) was used as a positive control. The scale bars are 20 μm. (**E**,**F**) The intracellular ROS levels were measured through staining by flow cytometric assay using dihydroethidium (DHE). Data are presented as means ± SD. All experiments were performed in three replicates. ** *p* < 0.01, *** *p* < 0.001 versus DMSO; # *p* < 0.05, ## *p* < 0.01 versus the group of FeSO_4_ alone.

As TfR1 performs a key role in cellular iron uptake, we next wanted to corroborate if concentrations of iron in cells were decreased by **12b**. The generation of Fe^2+^ ions was determined using a Fe^2+^ ions probe known as Ferrorange which reacts with Fe^2+^ ions to produce a bright fluorescent substance. Compared with the control groups, the orange fluorescence of cells treated with **12b** was much weaker (Figure 6A), suggesting **12b** attenuated the influx of Fe^2+^. The result suggests that TfR1 may be affected by **12b**.

It has been reported that Transferrin receptors are down-regulated at high concentrations of iron [49]. Therefore, we pre-used FeSO_4_ to reduce the level of TfR1 and to investigate the effect of **12b** on cell apoptosis. As expected, FeSO_4_ obviously decreased TfR1 expression. We found that **12b** also reduced the expression of TfR1 accompanied by an increase in the apoptosis protein C-PARP, which was attenuated after **12b** treatment combined with FeSO_4_ (Figure 6B,C).

Iron is involved in a number of cellular metabolic processes and the imbalance of iron homeostasis can directly or indirectly lead to the generation of ROS and induce cell damage or death [50]. To explore the effect of **12b** on the intracellular oxidative environment, the ROS generation capacity was assessed. We compared the ROS yielding ability of different groups by means of CLSM and flow cytometry. A fluorescent probe H_2_DCFDA was chosen to test intracellular ROS generation by CLSM. After incubation with **12b**, the fluorescence intensity of the cells became weaker in a dose-dependent manner (Figure 6D). Quantitative fluorescence analysis of DHE also demonstrated the result of attenuating intracellular ROS upon dosing with **12b** (Figure 6E,F). These results demonstrate that **12b** reduces the expression of TfR1, and subsequently impairs the influx of Fe^2+^ and with reduction of intracellular ROS generation, thus leading the cellular oxidative imbalance to cell apoptosis.

#### 3.2.6. Compound **12b**—Induced Apoptosis of MDA-MB-231 Cells Depends on TfR1

**Figure 7:**
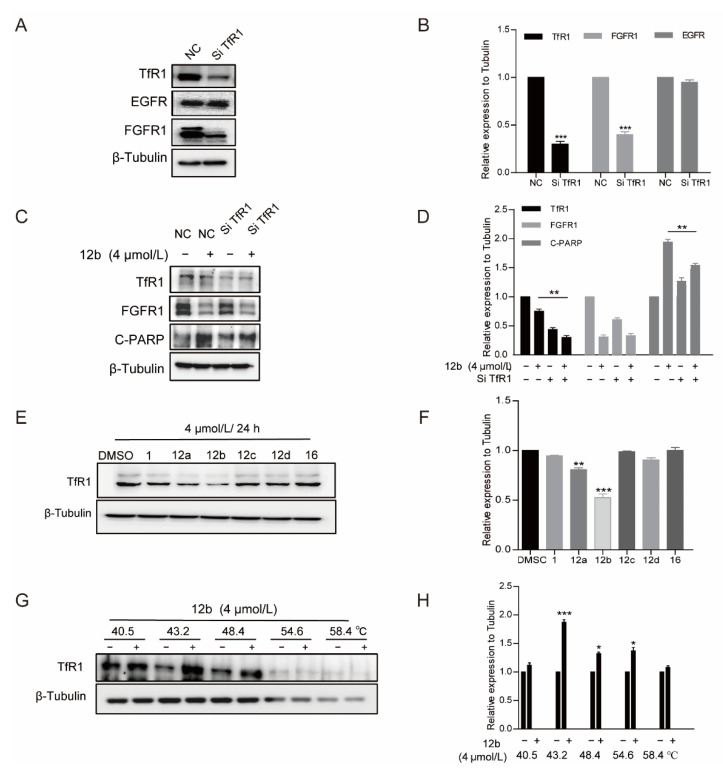
**12b**-induced apoptosis of MDA-MB-231 cells depends on TfR1. (**A**,**B**) MDA-MB-231 cells were transfected with TfR1-targeting siRNA by using Lipo3000 transfection reagents. After 48 h, the transfection efficiency was examined by Western blotting. (**C**,**D**) Cells with/without TfR1-knockdown were treated with **12b** (4 μM), protein levels were analyzed by Western blotting, and protein band densities were quantified by normalizing to β-Tubulin. (**E**,**F**) MDA-MB-231 cells were treated with **12a**–**12d** and **16** (4 μM) for 24 h, the level of TfR1 was detected by Western blotting, The relative band intensities of proteins to the β-Tubulin were analyzed and densities were quantified by normalizing to DMSO group. (**G**,**H**) Effect of **12b** on TfR1 stabilization. MDA-MB-231 cells were treated with or without **12b** (4 μM) for 2 h before being heated at different temperatures. Protein levels were analyzed by Western blotting. The relative band intensity to β-Tubulin was analyzed. Protein band densities were quantified by normalizing to DMSO. Data are presented as means ± SD. All experiments were performed in three replicates. * *p* < 0.05, ** *p* < 0.01, *** *p* < 0.001 versus DMSO.

Further, we treated MDA-MB-231 cells with TfR1-targeting siRNA to deplete its expression to investigate the pro-apoptotic activity of **12b**. It was observed that the expression of TfR1 was obviously decreased. Similarly, cells with TfR1 depletion produced less C-PARP than control cells upon **12b** treatment for 24 h. Interestingly, we noted that FGFR1 was down-regulated while EGFR had no significant change in TfR1 knocked down cells, which is consistent with the reduction of FGFR1 but not EGFR caused by **12b** in Figure 4B, supporting that TfR1 was affected by **12b**. These results indicate that **12b** required TfR1 to exert its activity (Figure 7A–D).

We then explored whether the difference in antiproliferative activity (Table 1) of compounds **12a**–**12d** and **16** in MDA-MB-231 cells is related to TfR1, thus the cell viabilities and the expressions of TfR1 were evaluated by MTT assay and Western blot after treatment of a series of compounds. As shown in Figure 7E, the antitumor activities of these compounds were in line with the inhibition abilities on TfR1 expressions, and **12b** reduced TfR1 expression most significantly accompanied by the most strong activity of proliferation inhibition. The data further indicate that **12b** exerts antitumor effects through TfR1.

Finally, we used the CETSA method to verify the binding between TfR1 and **12b** with the antibody of TfR1. As shown in Figure 7G, TfR1 was unstable with an increase in incubation temperature, which was reversed by **12b** treatment, corroborating that **12b** binds to TfR1 in MDA-MB-231 cells.

Together, these results demonstrate that TfR1 is a molecular target of **12b**, mediating the deficiency of iron metabolism and degradation of FGFR1, thereby inducing cell toward apoptosis in MDA-MB-231 cells.

## 4. Discussion

Triple negative breast cancer (TNBC) exhibits an aggressive subtype and a poor prognosis because these cancers lack ER, PR, and Her2 receptors [51]. Therefore, TNBC does not respond to hormone therapies or Her2 targeted therapies [52]. Given the paucity of effective targeted treatments for this disease subtype, this remains an intense area of investigation. TfR1 is highly expressed in TNBCs, particularly under iron-deficient conditions. Overexpression of TfR1 is thought to meet the increased requirements of iron uptake as well as intracellular ROS levels which are necessary for cell growth [53]. Together with its extracellular accessibility, ability to internalize, and central role in cancer cell pathology, TfR1 inhibition represents a promising anticancer strategy to treat TNBCs.

Muriceidine A, a new compound first isolated by us from the South China Sea gorgonian Muriceides collaris was a potent anticancer agent toward K562 cells with stronger cytotoxicity than that in HL60, A549, and Hela cells at an IC_50_ value of 8.37 μM [28]. However, its activity was not very satisfactory and the mechanism was also unclear. In the present paper, we evaluated the cytotoxicities of several derivatives with different substituent groups introduced in the functional unsaturated piperidine moiety of Muriceidine A in various cancer cells. It was shown that **12a**, **12b**, and **12c** showed stronger cytotoxicity against K562 cells, and the MDA-MB-231, Hela, and HCT-116 cell lines were sensitive to the three compounds. **12b** (methyl at position C-2 of Δ^1^-piperidine) exhibited the strongest cytotoxicity and induced cell apoptosis in MDA-MB-231 cells and structure-activity relationship analysis revealed that Δ1-piperidine was essential for antiproliferative activity and electron-donating groups at position C-2 of Δ^1^-piperidine was beneficial to anticancer activity.

The mechanism of the antitumor activity of **12b** was further examined. We found that **12b** treatment of MDA-MB-231 cells significantly reduced the phosphorylation of signal transduction proteins such as stat3, Scr, and Erk, which suggested to us that receptor tyrosine kinases (RTKs) may play a crucial role in **12b**. We found the levels of FGFR1, PDGFR, and VEGFR were down-regulated in a dose- and time-dependent manner except for EGFR. Furthermore, FGFR1 and PDGFR did not bind to **12b**. Therefore, we turned to other methods to look for a target molecule. Using a modified CESTA experiment with RCA I as a binding tag analyzed by LC-MS/MS protein spectrum, an obvious receptor molecule TfR1 was fished. Increasing evidence supports FGFR1, a kind of typical RTK, of which expression is an independent negative prognostic factor in TNBC [45,46,47,54]. FGFR1 was selected as an example and we found that whether FeSO_4_ or siRNA inhibits the expression of TfR1, the levels of FGFR1 were reduced accompanied by decreases in Fe^2+^ concentration and cell apoptosis. It was noticed that EGFR was not changed after TfR1 was deleted, which is in line with the **12b** treatment on MDA-MB-231 cells. Iron serves important functions for enzymes involved in DNA stability and cell cycle progression, ROS-generated enzymes in the electron transport chain, such as nicotinamide adenine dinucleotide phosphate hydride (NADPH) oxidase, P450 enzymes, lipoxygenases (LOX), and xanthine oxidase [55]. In line with these reports, **12b** displayed an arrested cell cycle at the S phase and reduced the generation of intracellular ROS due to blocking the uptake of Fe^2+^. These results suggest that TfR1 might be a target of **12b**. We further performed the CESTA experiment with the TfR1 antibody and it was shown that **12b** could bind to TfR1. Furthermore, the anti-proliferation activities of Muriceidine A derivatives **12a**–**12d** and **16** were proved consistent with the inhibition abilities on TfR1 expressions. Together, all these results corroborate that **12b** exerts antitumor effects through targeting against TfR1.

The mechanism of TfR1 involved in tumorigenesis remains poorly characterized and TfR1 has only been reported to act as a mitochondrial regulator to activate the JNK signaling pathway of lung cancer cells [56]. In our study, the findings that depletion of FGFR1 in TfR1 siRNA-treated cells and **12b**-induced down-regulation of FGFR1 are related to proteasome degradation suggest that TfR1 may be an upstream molecule of FGFR1 and promote the proteasome pathway degradation of FGFR1 after **12b** treatment in MDA-MB-231 cells. However, the detailed molecular mechanism is not clear, which needs to be discovered in future research.

In all, **12b** as a Muriceidine A derivative, was demonstrated to bind to TfR1 directly and cause iron deprivation as well as dysregulation of the intracellular oxidative environment, inducing degradation of FGFR1 oncoprotein through proteasome pathway and TfR1-related cell apoptosis in MDA-MB-231 breast cancer cells. These results support that **12b** is a novel, promising, small molecule led compound targeting TfR1 against triple negative breast cancer cells worth further studying in the future, and the finding of the structure-activity relationship helps to design new more effective compounds against TfR1.

## 5. Conclusions

In this study, we have designed and synthesized a variety of new Muriceidine A derivatives and carried out systematic structure-activity relationship research. Most of the target derivatives showed stronger cytotoxicity than their parent compound, Muriceidine A, against the MDA-MB-231, K562, Hela, and HCT-116 cell lines. Particularly, we found that introduction of electron-donating groups at position C-2 of Δ^1^-piperidine was beneficial to anticancer activity. Additionally, the replacement of Δ^1^-piperidine with other unsaturated nitrogen heterocycle resulted in significantly decreased potency, suggesting that Δ^1^-piperidine was responsible for the antiproliferative activity. Among these compounds, **12b** (methyl at position C-2 of Δ^1^-piperidine) exhibited the strongest cytotoxicity against MDA-MB-231 cells and was more potent than Muriceidine A with an IC_50_ value of 1.15 μM. **12b** was demonstrated to bind to TfR1 directly and cause iron deprivation as well as dysregulation of the intracellular oxidative environment, in turn inducing degradation of FGFR1 oncoprotein through proteasome pathway and TfR1-related cell apoptosis in MDA-MB-231 breast cancer cells. These results support that **12b** is a novel promising small molecule lead compound targeting TfR1 against triple negative breast cancer cells worth further studying in the future.

## 6. Patents

There is a patent resulting from the work reported in this manuscript. Public Announcement No.: CN110791543A.

## Figures and Tables

**Table 1:** Antitumor activity of target compounds **12a**–**d** and **16** against four human cancer cell lines.

Compounds	IC_50_ (μM) ^a^			
	MDA-MB-231 ^b^	K562 ^c^	Hela ^d^	HCT-116 ^e^
**12a**	13.93 ± 0.042	6.99 ± 0.035	8.73 ± 0.016	>100
**12b**	**1.15 ± 0.010**	2.41 ± 0.029	1.21 ± 0.010	4.63 ± 0.041
**12c**	4.64 ± 0.017	1.91 ± 0.014	2.58 ± 0.020	3.22 ± 0.012
**12d**	10.91 ± 0.021	58.41 ± 0.043	>100	>100
**16**	>100	>100	>100	>100
Muriceidine A	77.61 ± 0.063	8.37 ± 0.070	17.97 ± 0.024	>100
Doxorubicin ^f^	0.50 ± 0.040	0.39 ± 0.010	0.55 ± 0.027	0.61 ± 0.080

^a^ Cell viability was determined by MTT assay (IC_50_ mean ± SD). ^b^ Human triple negative breast cancer cell line. ^c^ Human chronic myeloid leukemia cell line. ^d^ Human ovarian cancer cell line. ^e^ Human colon cancer cell line. ^f^ Doxorubicin was used as the positive control. The bold value shows the strongest antitumor activity of all these compounds in the four types of cells.

**Table 2:** Mass spectrometry analysis of proteins (scores > 80) interacting with **12b**.

Accession	Score *	Mass	Matches †	Sequences ‡	emPAI §
sp|P38646|GRP75_HUMAN	657	73,920	68 (35)	40 (28)	3.01
sp|P23588|IF4B_HUMAN	586	69,167	63 (33)	30 (17)	1.31
sp|Q96G03|PGM2_HUMAN	479	68,754	62 (28)	39 (20)	1.93
sp|P18206|VINC_HUMAN	386	124,292	39 (23)	27 (19)	0.68
sp|Q14247|SRC8_HUMAN	285	61,720	57 (23)	31 (15)	1.42
sp|Q15291|RBBP5_HUMAN	251	59,800	30 (10)	20 (9)	0.62
sp|Q09666|AHNK_HUMAN	218	629,213	44 (10)	38 (10)	0.05
sp|P31040|SDHA_HUMAN	186	73,672	36 (14)	21 (13)	0.84
sp|Q86YZ3|HORN_HUMAN	177	283,140	17 (9)	15 (9)	0.11
sp|P02545|LMNA_HUMAN	136	74,380	15 (9)	11 (8)	0.41
**tr|G3V0E5|TfR1_HUMAN**	**116**	**76,259**	**18 (4)**	**11 (3)**	**0.13**
sp|P10515|ODP2_HUMAN	101	69,466	20 (7)	15 (7)	0.38
sp|P23229|ITA6_HUMAN	96	127,724	9 (5)	8 (5)	0.13
sp|O95810|CAVN2_HUMAN	82	47,202	10 (6)	10 (6)	0.5
sp|Q912a23|CR025_HUMAN	80	43,597	8 (4)	4 (3)	0.24
sp|Q96HN2|SAHH3_HUMAN	80	67,705	11 (2)	8 (2)	0.1

* Score: A description of the correlation of this candidate protein. † Matches: Number of high confidence images of mass spectrometry (confidence interval > 95%). ‡ Sequences: Number of highly reliable peptides matched to the compound. § emPAI: Protein content. Bold values are parameters of TfR1 in mass spectrometry analysis.

## Data Availability

Data is contained within the article.

## Supplementary Materials

The following supporting information can be downloaded at: https://www.mdpi.com/article/10.3390/antiox11050834/s1, Figure S1: ^1^H-NMR spectra of compound Muriceidine A; Figure S2: ^1^H-NMR spectra of compound **12a**; Figure S3: ^13^C-NMR spectra of compound **12a**; Figure S4: ^1^H-NMR spectra of compound **12b**; Figure S5: ^13^C-NMR spectra of compound **12b**; Figure S6: ^1^H -NMR spectra of compound **12c**; Figure S7: ^13^C-NMR spectra of compound **12c**; Figure S8: ^1^H-NMR spectra of compound **12d**; Figure S9: ^13^C-NMR spectra of compound **12d**; Figure S10: ^1^H-NMR spectra of compound **16**; Figure S11: ^13^C-NMR spectra of compound **16**.
