# Supplementary material for: Identification of a Novel Inhibitor of TfR1 from Designed and Synthesized Muriceidine A Derivatives
Source: Antioxidants (Basel). 2022 Apr 25;11(5):834. doi: 10.3390/antiox11050834 (PMC9137542; doi:10.3390/antiox11050834)
Supplement: Supplementary file 1 [file antioxidants-11-00834-s001.zip › antioxidants-1671468-supplementary.pdf]

Supplementary Material

Identification of a novel inhibitor of TfR1 from designed and synthesized Muriceidine A derivatives

Yu Wu [1,⊥], Zongchen Ma [1,⊥], Xiaoyuan Mai [1], Xiaoling Liu[1], Pinglin Li [1], Xin Qi[1], Guoqiang Li [1,2,*] and Jing Li [1,2,*]

[1] Key Laboratory of Marine Drugs, Chinese Ministry of Education, School of Medicine and Pharmacy, Ocean University of China, Qingdao 266003, P. R. China

[2] Laboratory for Marine Drugs and Bioproducts, Open Studio for Druggability Research of Marine Natural Products, Qingdao National Laboratory for Marine Science and Technology, Qingdao 266237, P. R. China.

***Corresponding Author**

E-mail address: lijing_ouc@ouc.edu.cn (J. L.) and liguoqiang@ouc.edu.cn (G. L.)

[⊥]These authors contributed equally to this work

1H-NMR spectra and 13C-NMR spectra of compounds **Muriceidine A**. **12a-12d** and **16**

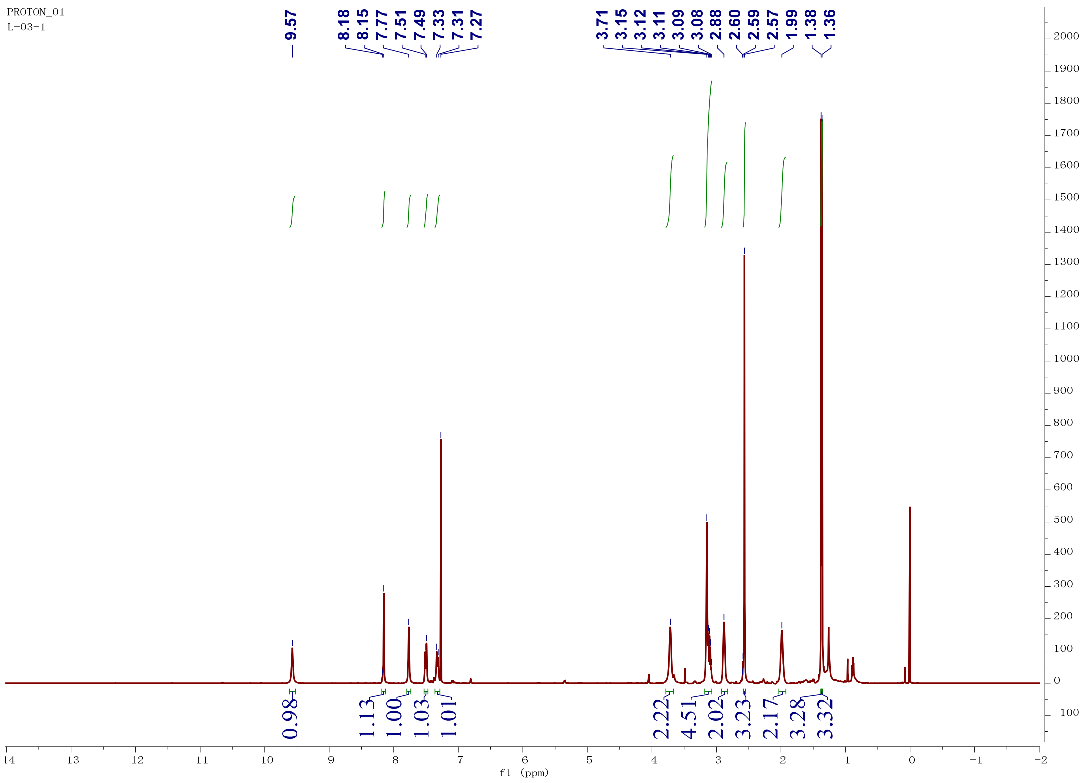

Figure S1: The ^{1}H-NMR spectra of **Muriceidine A**

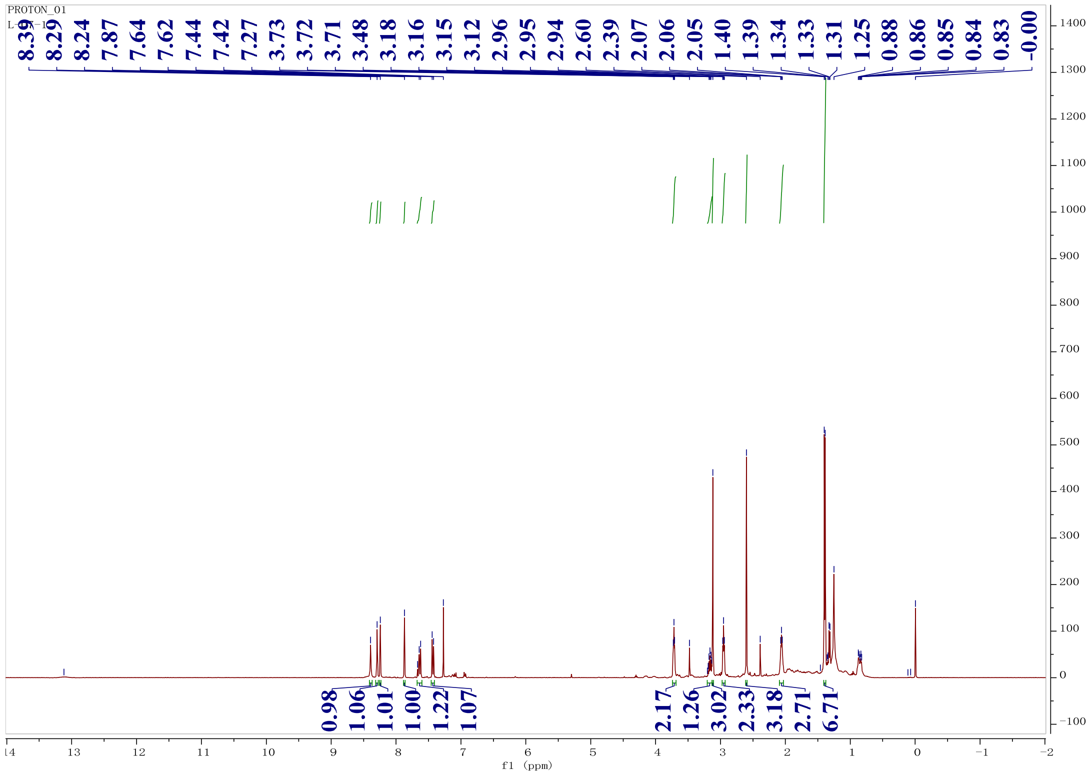

Figure S2: The ^{1}H-NMR spectra of **12a**

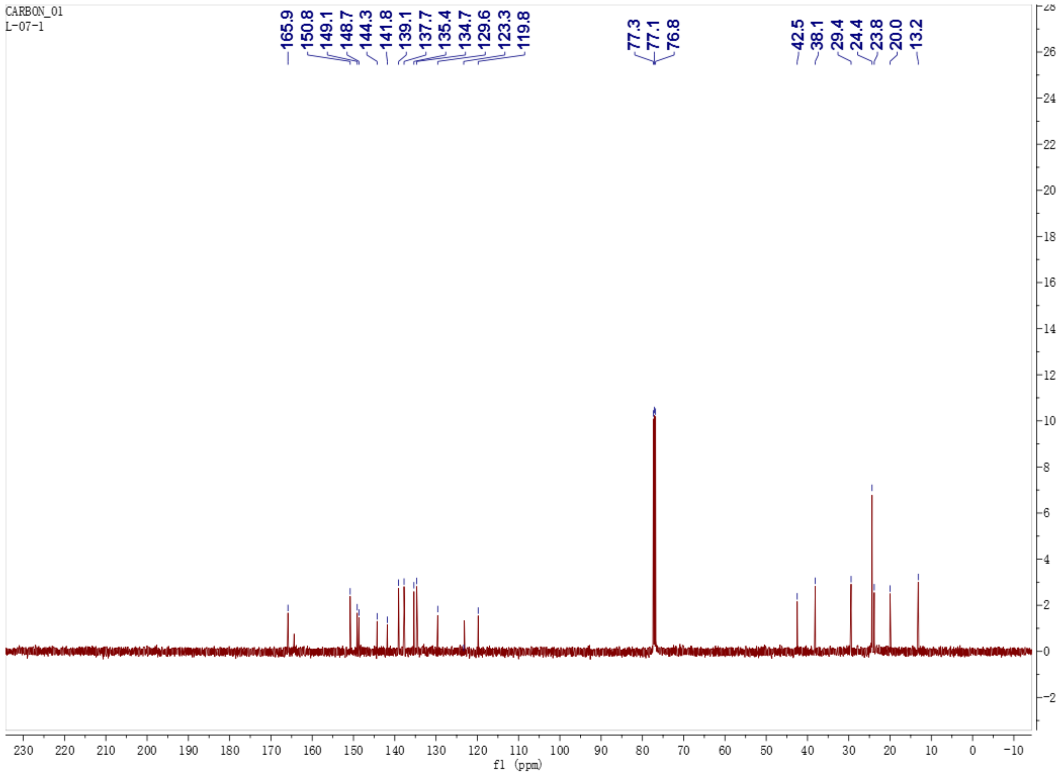

Figure S3: The ^{13}C-NMR spectra of **12a**

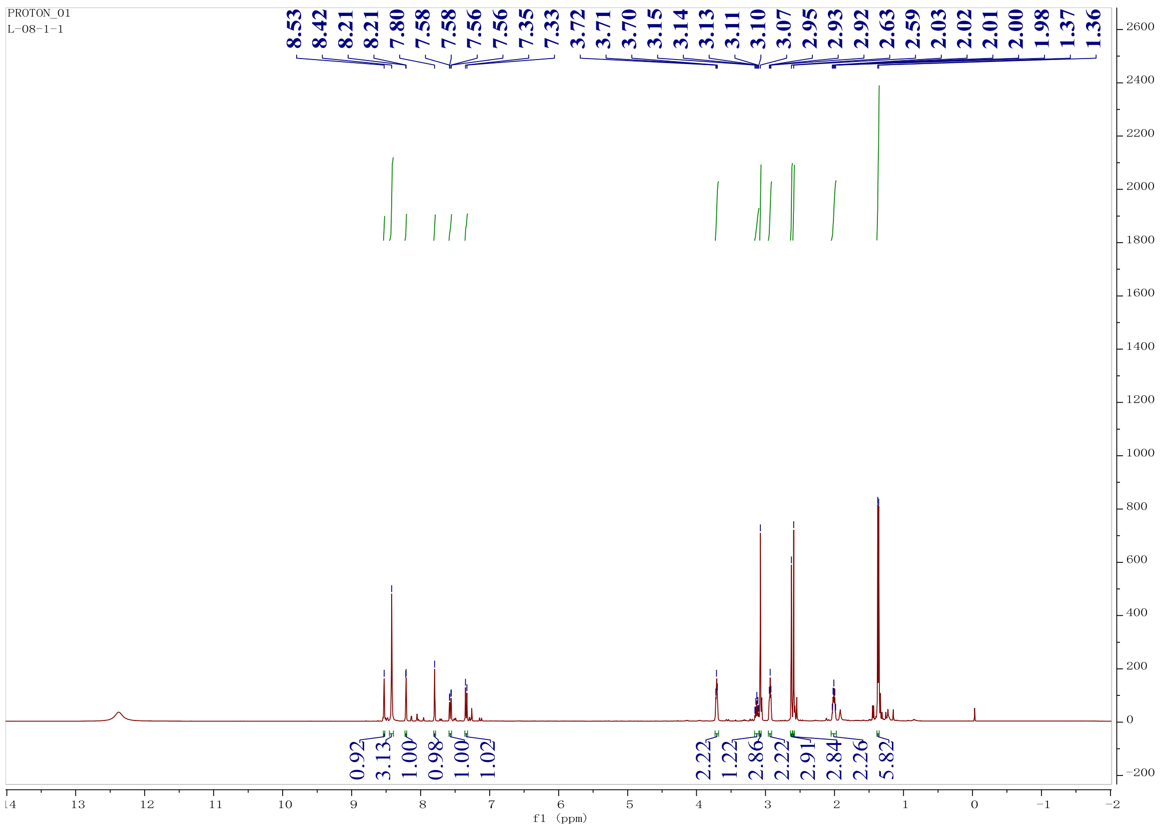

Figure S4: The ^{1}H-NMR spectra of **12b**

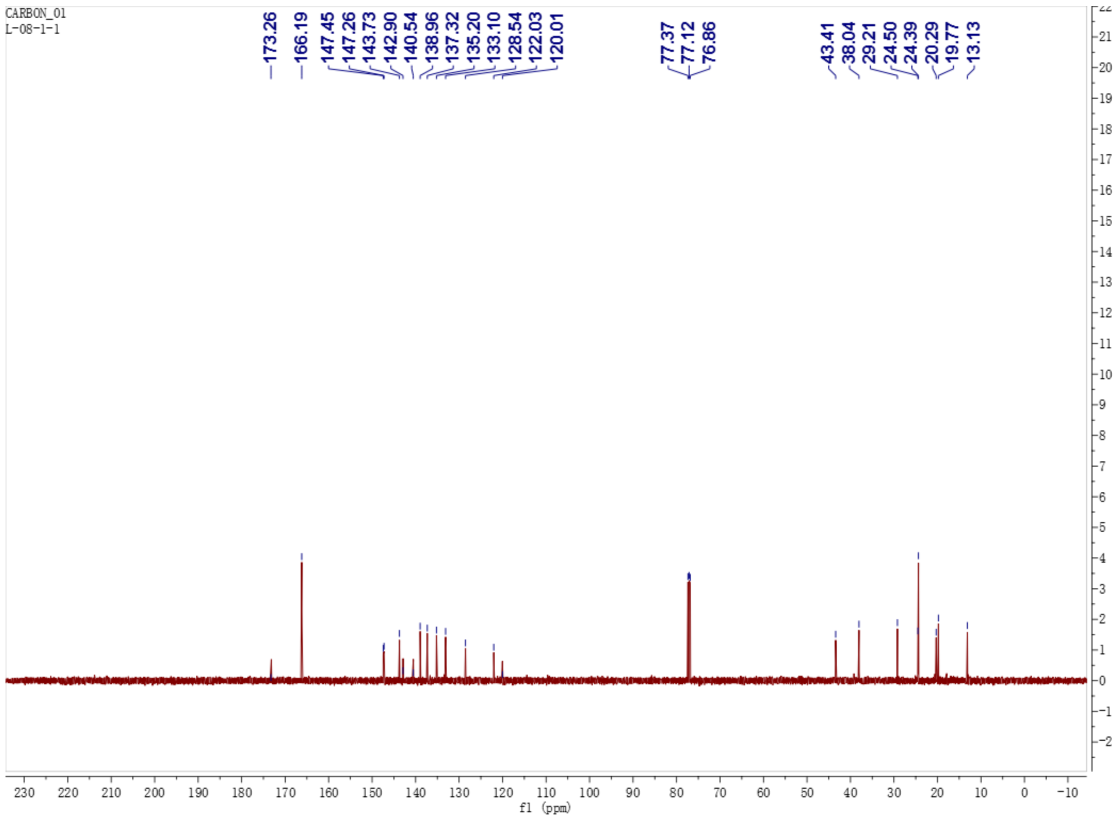

Figure S5: The ^{13}C-NMR spectra of **12b**

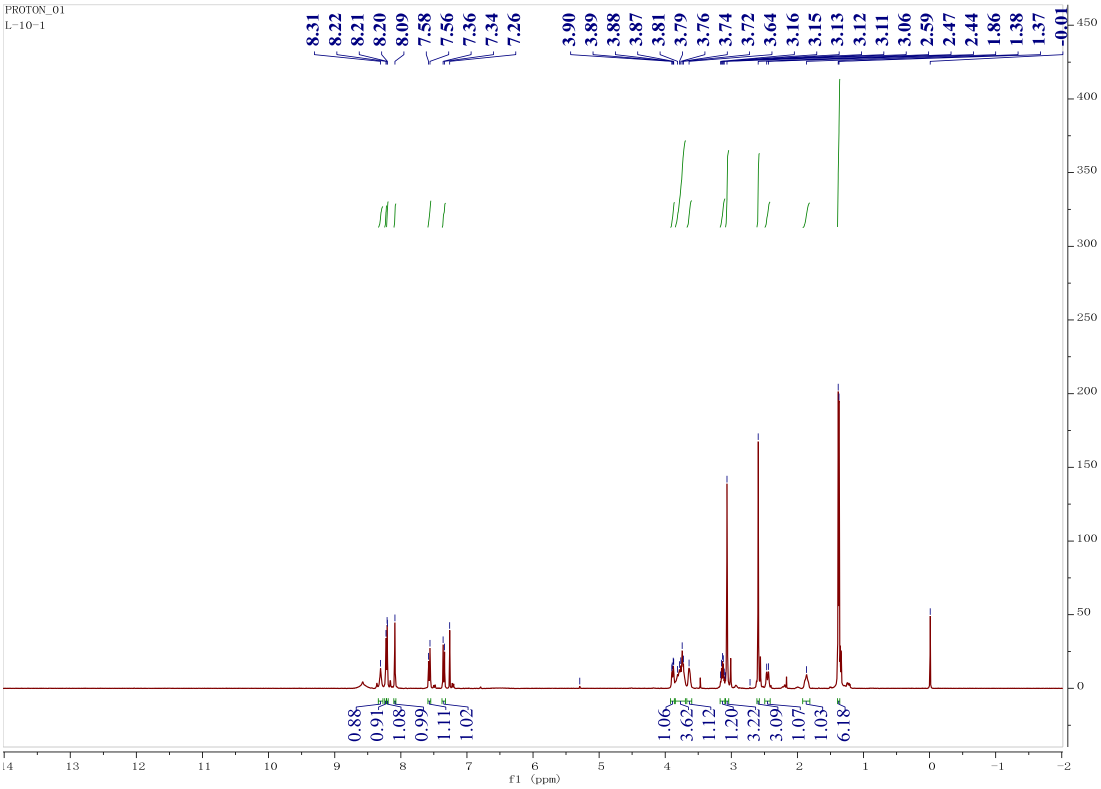

Figure S6: The ^{1}H-NMR spectra of **12c**

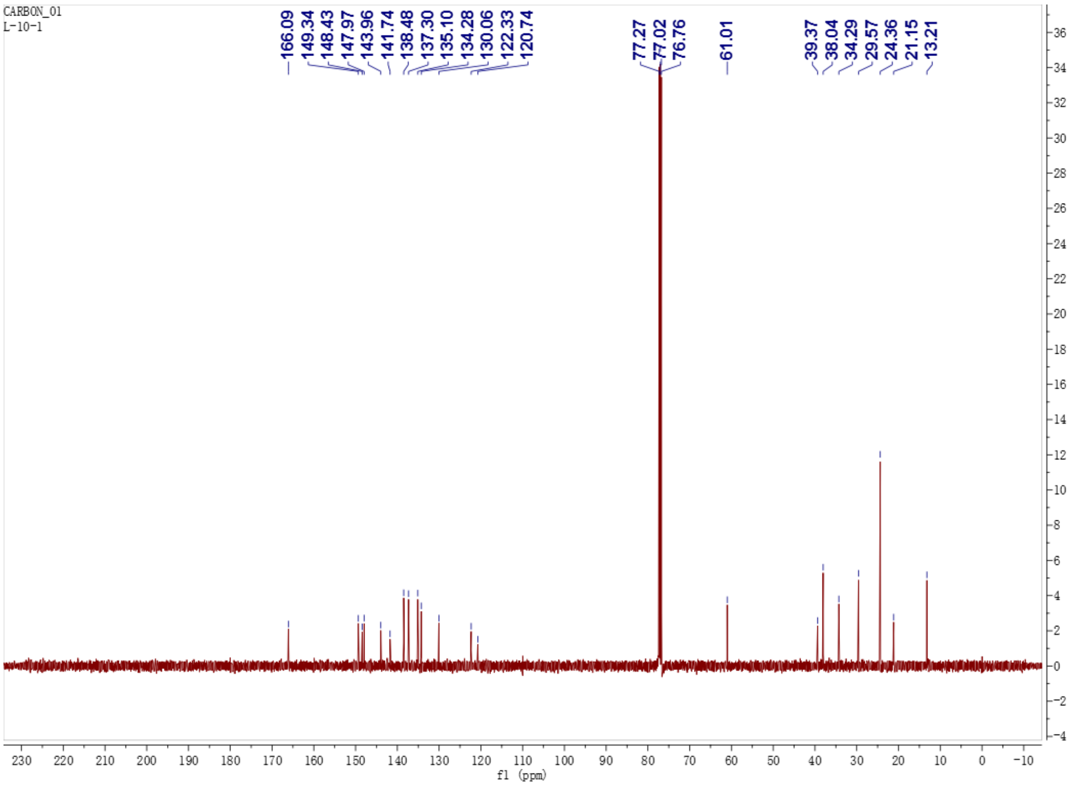

Figure S7: The ^{13}C-NMR spectra of **12c**

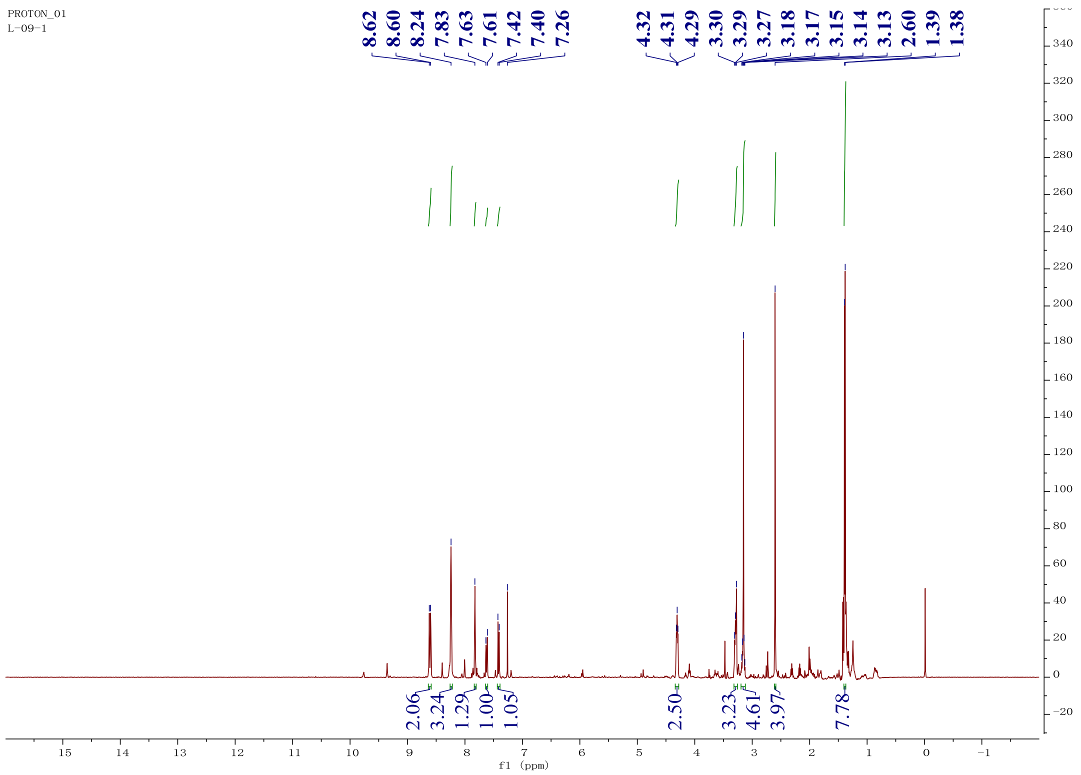

Figure S8: The ^{1}H-NMR spectra of **12d**

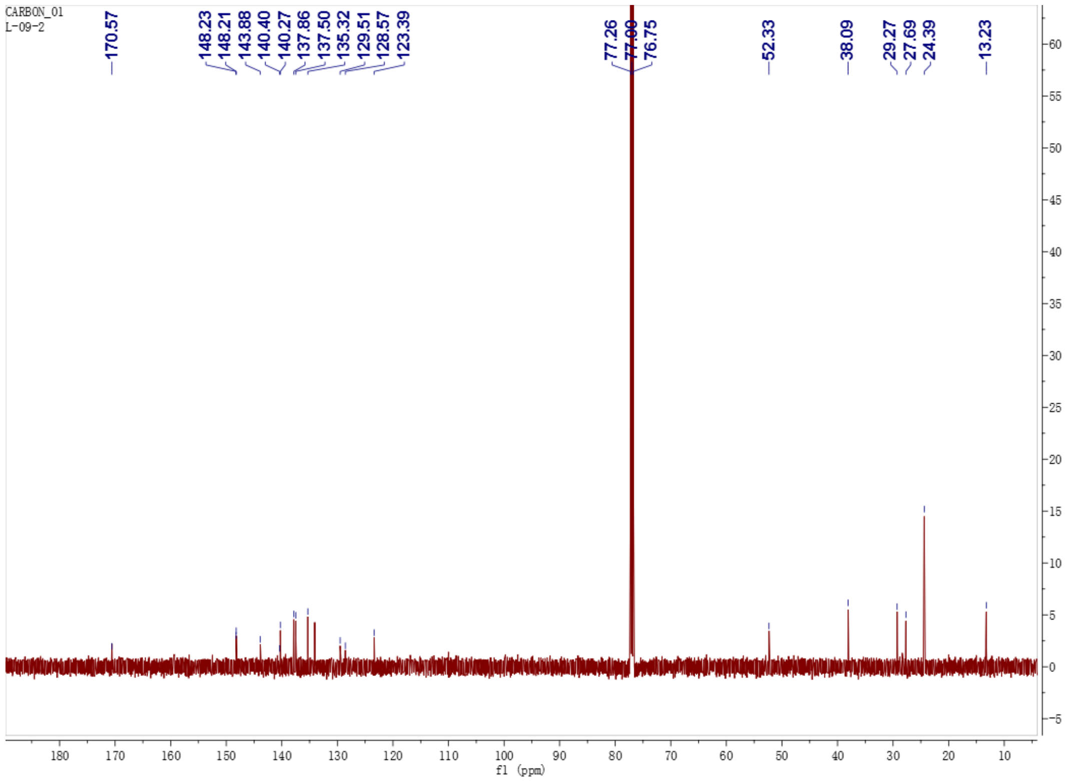

Figure S9: The ^{13}C-NMR spectra of **12d**

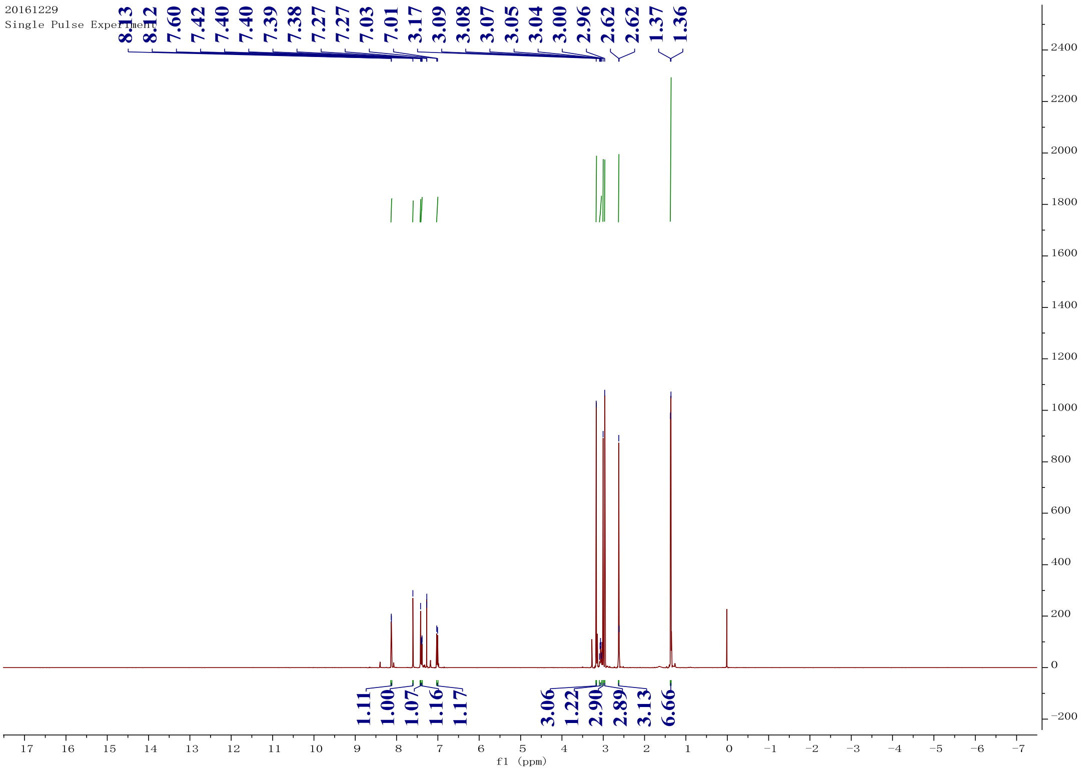

Figure S10: The ^{1}H-NMR spectra of **16**

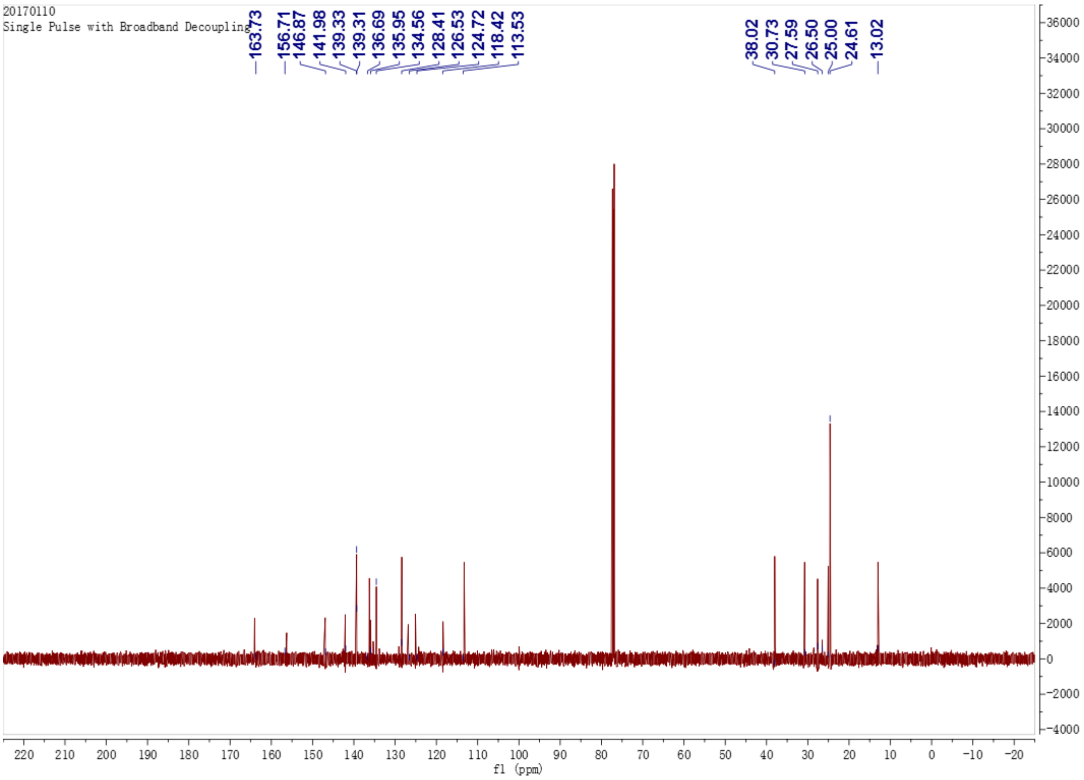

Figure S11: The ^{13}C-NMR spectra of **16**
